# Restenosis and Therapy

**DOI:** 10.1155/2012/406236

**Published:** 2012-02-23

**Authors:** Laszlo Denes, Laszlo Entz, Veronika Jancsik

**Affiliations:** ^1^Department of Pharmacology and Pharmacotherapy, Semmelweis University, Budapest 1089, Hungary; ^2^Department of Vascular Surgery, Semmelweis University, Budapest 1122, Hungary; ^3^Department of Anatomy and Histology, Faculty of Veterinary Science, Szent Istvan University, Budapest 1078, Hungary

## Abstract

The vascular disease involves imbalanced function of the blood vessels. Risk factors playing a role in development of impaired vessel functions will be briefly discussed. In ischemia/reperfusion (I/R), ischemic hypoxia is one of the cardinal risk factors of restenosis. Various insults are shown to initiate the phenotype switch of VSMCs. The pathological process, leading to activated inflammatory process, complement activation, and release of growth factors, initiate the proliferation of VSMCs in the media and cause luminal narrowing and impaired vascular function. The review summarizes the alteration process and demonstrates some of the clinical genetic background showing the role of complement and the genotypes of mannose-binding lectin (MBL2). Those could be useful markers of carotid restenosis after stent implantation. Gene therapy and therapeutic angiogenesis is proposed for therapy in restenosis. We suggest a drug candidate (iroxanadine), which ensures a noninvasive treatment by reverse regulation of the highly proliferating VSMCs and the disturbed function of ECs.

## 1. Introduction

A luminal narrowing in the arterial vessel wall is induced by injury that is leading to stenosis and development of intimal hyperplasia [1]. The balloon coronary angioplasty has represented a revolutionary treatment that led to the birth of interventional cardiology. This new special surgery process with the exclusion surgical intervention produces ischemia reperfusion, inflammation and final to restenosis with an occlusion of arterial lumen. Proven indications for endarterectomy as recoil are moderate-to-high grade stenosis, rapidly progressive stenosis with lesions revealing greater than 70 percent in diameter of stenosis in carotid diseases. Clinical results reveal primary success rates, less complication, and restenosis rates comparable with those of surgical endarterectomy. The coronary stents, which were first developed in the mid-1980s, have ultimately replaced the “old balloon angioplasty” as the preferred method of performing percutaneous transluminal coronary angiography (PTCA) intervention [2]. After the observed improvements (in angiographic and clinical outcomes), this field represents the most common major worldwide medical procedure. Several groups have been published the similar results like as our group. 171 interventions on 151 patients were performed as a single surgeon's experiences and long-term follow-up was completed in 109 patients. The combined survival rate was 85%, recurrent stenosis-free 88% at 5 years, respectively. Only 9% of the patients had carotid restenosis >70% [3].

Reduction of the incidence of clinical events was only possible if a low perioperative complication rate was accomplished. Nowadays involve a coronary stent, and interventional cardiologists are faced with a wide choice of coronary stents to implant, such as bare metal stents and drug-eluting stents. Coronary stenting only became a widely accepted technique [4]. In spite of some relevant strong points such as greater than 90% success rate and the repeatability of the procedure, several major drawbacks still persist, including restenosis within the treated vessel [5–8]. The restenosis phenomenon is currently the object of intensive research in different areas of biomedical field and therapy.

Restenosis means the reoccurrence of stenosis, a narrowing of a blood vessel, leading to restricted blood flow. Restenosis usually sets to an artery or other large blood vessel that has become narrowed. This phenomenon is grown up after balloon intervention with an enhanced inflammatory processes and the blood vessel subsequently becomes renarrowed. This term is common in vascular surgery, cardiac surgery, and angioplasty, all branches of medicine that frequently treat narrowing of blood vessels [9]. Restenosis can be defined as a reduction in the circumference of the lumen of 50% or more and had a high incidence rate (25–50%) in patients who had undergone balloon angioplasty, with the majority of patients needing further angioplasty within 6 months [10].

Damage to the blood vessel wall by angioplasty triggers physiological response that occurs immediately after tissue trauma, is thrombosis. A blood clot forms at the site of damage and further hinders blood flow with an inflammatory response. The second stage tends to occur 3–6 months after surgery and is the result of proliferation of cells in the intima, a smooth muscle wall in the vessel. This is also known as early restenosis, namely, neointimal hyperplasia [11]. The artery can react to the stent, perceive it as a foreign body, and respond by mounting an immune system response (see physiology below) which leads to further narrowing near to or inside the stent. Drug-eluted stents substantially reduce the occurrence of restenosis but the clinical studies indicate a slight incidence rate of recurrence (≥5% ) [12]. Obviously restenosis remains a problem.

Restenosis is mainly due to neointima formation, which is caused primarily by the effects of VSMC proliferation and migration [12]. Cell proliferation after stenting occurs both early, as part of the acute injury response, and late, located around stent struts. Whereas some neointima formation is necessary for vessel healing after stenting, excessive neointima formation narrows the lumen [13]. The function of endothelium plays a central role in destroyed balance and later the maintaining of vascular health by virtue of the endothelial cells [14]. Leakage of the endothelium lining and prolonged endothelial proliferation is characterized after the intervention [15]. Development of restenosis has demonstrated high proliferation activity of VSMC from the tunica media to the intima which is the occurrence of internal elastic lamina rupture [16, 17]. To summarize, the adverse cardiovascular events are associated to endothelial dysfunction and activated proliferation of VSMC [18].

Atherosclerosis is coexistence with occlusion of arterial wall and the etiology of the disease distinct of restenosis. It is known as vascular disease in which an artery wall thickens as a result of the accumulation of cholesterol. It is a chronic inflammatory response, caused largely by the accumulation of macrophages and lymphocytes and promoted by high level of oxidized low-density lipoproteins [19]. It is commonly referred to as a hardening of the arteries and caused by the formation of multiple plaques within the arteries. The advanced atherosclerosis is chronic, slowly progressive, and cumulative process. Most commonly, the soft plaques suddenly create ruptures [20] causing the formation of a thrombus and incidentally leading to death of the tissues (infarction).

The most important risk factors of MIH and restenosis are ischemia/reperfusion injury, shear stress, inflammation, diabetes, oxidative stress, hypertension, modulation of cytokine, and C-reactive protein [CRP] level, together with other environmental stimuli such as smoking. Those factors which appear to have impact on the evaluation of restenosis will be briefly mentioned in this review.

### 1.1. Ischemia/Reperfusion (I/R) Injury

I/R injury plays a significant role in the pathophysiology of restenosis by inducing endothelial dysfunction. Ischemia induces rapid and gradual injuries in cells of blood vessels. The arterial blood vessel is vulnerable to I/R injury which depends on the ischemic time, hemodynamic status of patients, and the reperfusion itself. I/R injury of endothelial cells (ECs) apparently provide an initial trigger and subsequently causes enhanced proliferation of VSMCs. Thus, diminution of I/R injury would be a great benefit not only by inhibiting direct cellular injury but also indirectly through the mentioned factors that influence several processes, for example, the immune response [21]. The injury can block the artery or result in poor perfusion of reperfused tissue of blood vessel. PTCA causes a significant inflammatory response compared to angiography alone. Ischemia/reperfusion injury correlated with restenosis and inflammatory response. Time-dependent increased level of IL-6 and TNF-alpha has detected in PTCA patients [22].

### 1.2. Shear Stress

Vascular homeostasis on arterial walls is highly dependent on the blood flow and shear stress. Responses to shear stresses (laminal and oscillatory) are well summarized [23]. Endothelial cells act as sensors of shear stress and regulate its level by adapting the arterial dimensions to blood flow. Shear stresses control and modify function of cells in arterial wall. In the presence of several risk factors, low shear stress contributes to endothelial dysfunction, whereas normal-to-high shear stress results in protecting the function of blood vessel. High shear stress upregulates the expression of endothelial genes and proteins which are protected against dysfunction, and by low shear stress an opposite effect is produced. The role of shear stress in restenosis is less understood [23]. Disturbed flow has been proven to result in postsurgical neointimal hyperplasia, in-stent stenosis, and aortic valve calcification [24].

### 1.3. Immune Factors

The immune system plays important roles in restenosis. Endothelial injury and activation elicits the release of proinflammatory cytokines, chemokines, and expression of adhesion molecules, which fosters immune cell recruitment and transmigration of immune cells across the EC barrier and into the intima. The role of various immune components in restenosis is complex. The current review will not delve into details regarding the role of immunity in development of restenosis [25–27].

### 1.4. Thromboembolism

Thromboembolism, governed by several risk factors (hypertension, diabetes, obesity, smoking, etc.) constitutes an increased risk and complication of hospitalization. The phenomena of thromboembolism overlap with the phenomena of atherothrombosis and/or restenosis [28].

### 1.5. Elevated Serum C-Reactive Protein Level

Elevated C-reactive protein (CRP) level associated with inflammation may promote the restenosis process. Exogenous CRP induces the expression of adhesion molecules [29] and decreases endothelial NOS (eNOS) production [30]. Furthermore, CRP upregulates SMC angiotensin-I receptors and thereby increases the level of the reactive oxygen species as well as proliferation [31]. In addition, monocytes/macrophages exposed to CRP increase the release of tissue factor, which potentially stimulates cell migration and adhesion to endothelial cells [32] extending inflammatory process and finally results in restenosis.

### 1.6. Hyperhomocysteinemia

Epidemiological evidence shows that high level of serum homocysteine is associated with an increased risk of cardiovascular diseases. Hyperhomocysteinemia affects primarily the endothelium, which in turn results in reduced endothelial NO production, decreased arterial response to vasodilators, and increased expression of procoagulant factors. Normalization of serum homocystein level might be an effective therapeutic approach [33].

### 1.7. Further Risk Factors

Among others, hypertension, smoking, and diabetes mellitus are risk factors associated to the development of restenosis. Hypertension causes endothelial injury by promoting the formation of intimal hyperplasia [2]. In spite of our increasing knowledge on the nature and effect of numerous risk factors, partially covered above, further studies are clearly needed to establish important features of their mode of action as well as their interactions in the pathogenesis of myointimal hyperplasia and restenosis. We suggest that I/R injury and hypoxia are one of the initiative risk factors of restenosis, acting by disturbing the normal functioning of the endothelial monolayer. As a result, endothelial cells release enhanced levels of inflammatory cytokines and growth factors, leading to phenotype switch of the vascular smooth muscle cells, followed by their increased migration and/or proliferation. This suggestion will be further developed below.

## 2. Cellular and Physiological Events during Restenosis

### 2.1. Endothelial Cells

The endothelium regulates many important functions in the cardiovascular system including vascular tone, coagulation, and inflammatory responses. In addition, the endothelium limits local thrombosis by producing tissue plasminogen activator, maintaining a negatively charged surface, and secreting heparans, NO, and thrombomodulin [34].

NO, a paracrine factor produced by the endothelial NO synthase enzyme (eNOS) is well known as vasodilator [35]. In addition, its effect is crucial for several other processes as well, involving the prevention of platelet aggregation/adhesion and reduction of the expression of several proinflammatory genes. Pharmacological inhibition or genetic deficiency of eNOS hampers endothelium-dependent vasodilatation, impairs tissue blood flow, and raises the blood pressure [36].

NO plays a key role in maintaining vascular homeostasis, by exerting a wide variety of effects on the endothelial and smooth muscle cells of the vessel wall. It stimulates endothelial cell proliferation, mainly by potentiating the mitogenic effect of vascular endothelial growth factor. On the contrary, in the case of VMSCs, NO inhibits their proliferation and migration. As a consequence, enhanced NO production in the vessel wall slows down or even reverses restenosis [37, 38].

Beside NO, mitogens (e.g., PDGF) produced by ECs are also important regulators of VSMCs. Selective inhibition of PDGF production by low-density lipoprotein has potentially profound implications for the clinical control of MIH [39].

In summary, endothelial dysfunction (mechanical injury to the endothelium) is an important early feature of vascular injury with serious impact on the pathogenesis of restenosis.

### 2.2. Vascular Smooth Muscle Cells and Phenotype Switch

Induced proliferation of VSMCs contributes to the pathobiology of restenosis and linked also to other cellular processes such as inflammation, apoptosis, and matrix alterations. In fact, beside inflammation enhanced proliferation of VSMCs is one of the key processes of restenosis and atherosclerosis [40].

**Figure 1:**
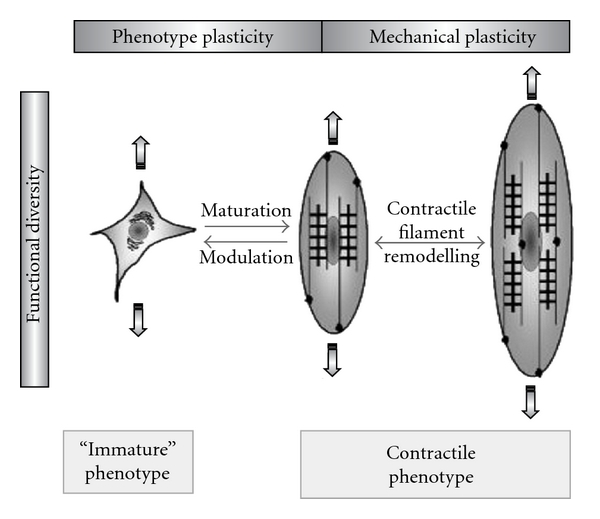
Correlation of phenotype, mechanical and functional plasticity of VSMCs (figure from [41]).

Enhanced proliferation of VSMCs is most probably the consequence of phenotype switch of these cells. Phenotype switch is a complex cellular mechanism providing the possibility of adult smooth muscle cells to fluctuate reversibly between a contractile and an “immature” (also called “proliferative”) phenotype. In response to various inner and environmental signals, these two phenotypes exert different activities: contractile smooth muscle cells stiffen, shorten and relax, whereas the immature ones have a tendency to proliferate, to migrate, and to synthesize extracellular matrix components. It is to be noted, that within both phenotypes functional diversity occurs, that is, distinct populations might coexist which respond differently to given stimuli [41]. This is schematized in Figure 1.

Molecular mechanisms underlying the functional plasticity of the contractile phenotype are centered on (a) remodeling of the actomyosin filaments constituting the contractile apparatus [42], and (b) variation of the expression of proteins regulating cell responses to environmental cues. Numerous proteins belong to this latter group, involving receptors, signaling effectors, ion channels, and so forth, preferentially sequestered in caveolae, flask-shaped invaginations of the plasma membrane. Caveolae are of primary importance in regulating the phenotype switch of VSMCs [43]. Caveolae components are differently organized in the distinct myocyte phenotypes; they are more abundant in contractile vascular myocytes [44]. In contractile VSMCs they regulate processes leading to functional plasticity, and they are suggested to be involved the integration of events leading to phenotype switch.

At least two transmembrane adhesion receptors, the integrins and the dystrophin-glycoprotein complex (DGC) are present in caveolae, which constitute a link between the extracellular matrix (ECM) components, (e.g., laminins) and the intracellular actin filament system, being therefore key players in the communication of the cells with their environment. Noticeably, different types of integrins and DGC components are expressed in VSMCs of different phenotypes (for a review, cf. [45]). 

In the course of restenosis, one of the first steps of the lesion development is the focal accumulation of VSMCs within the intima. The local inflammatory milieu can induce expression of collagenase and inhibit expression of proteolytic inhibitors [46]. In advanced lesions, fibroblasts and VSMCs with extracellular calcification form a fibrocalcific plaque. The origin of VSMCs in the atherosclerotic plaque is controversial. The VSMC population in intimal lesions has been proposed to arise from medial VSMCs [47], adventitial cells [48], preexisting intimal clones [49], and precursor cells derived from the vessel wall itself or from circulating vascular progenitors [50]. Several factors have been found to affect VSMCs. Principal factors among these are platelet-derived growth factor (PDGF), somatomedin-C, epithelial growth factor, and insulin. Lipoproteins may be also important substrates for VSMC proliferation, while heparin directly inhibits VSMC protein synthesis [51, 52]. Internal lesion of arterial wall EC results in subsequent proliferation of VSMCs. The degree of VSMC proliferation appears to be dependent mainly on the degree of initial injury and partially, but not in all cases on loss the confluence of overlying endothelium. Heparin and other EC products appear to inhibit VSMC proliferation independently of platelet-VSMC interaction. Platelets may play a role in the early response to arterial injury and development of MIH, but their long-term role appears to be minor [52, 53]. Hypertension results in a marked proliferation of VSMC enhancing the proliferative response to injury.

Structural organization and signaling of VSMCs and thereby phenotype switch are influenced by externally applied mechanical force as well. The predominant mechanical force acting this way is cyclic stretch, which regulates the activity of their contractile apparatus. Drug therapy directed at the components of the signaling pathways influenced by stretch may prevent myointimal hyperplasia and thereby restenosis [54].

Recently, we have discovered that hypoxia; one of the cardinal risk factors of restenosis influences both the endothelial monolayer and the VSMCs. These cell types are inverse regulated by a single short duration of hypoxia. Endothelial cells undergo apoptosis, whereas the proliferative activity of VSMCs increases significantly [55]. The contractile phenotype of VSMCs is changed to proliferative type.

## 3. Clinical Features of the Restenosis

### 3.1. Clinical Analysis and Diagnostic Markers

In the last decades surgery techniques have been routinely performed [2, 56]. The restenosis is one of the most common and dangerous postoperative complication of these interventions. Pathology of coronary diseases in general comprises several distinct features involving, among others, degenerative and cell proliferation/differentiation processes. Cell proliferation and differentiation disturb the cellular morphogenesis [57] leading to the formation of a multilayered compartment internally to the elastic membrane of the arterial wall, composed of cells expressing alpha-smooth-muscle actin [58]. This process is termed as “arterial intimal hyperplasia” “myointimal hyperplasia,” or “neointima formation,” and it is the leading pathological mechanism leading to the narrowing of the arterial lumen. Arterial intimal hyperplasia is one of the main factors of coronary artery diseases [59]. It is destructive event of the postoperative complications after angioplasty, bypass operations, or stenting as well [60, 61]. Several attempts have been made to interfere with the development of postoperative intimal hyperplasia, of which application of drug-eluting stents (DES) seems to be the most promising approach [57, 62].

Our vascular surgeon team has been performing endovascular interventions since decades. In a comprehensive retrospective study [3, 61], it was shown that the 5-year patient survival rate after carotid endarterectomy (CEA) was 85%, and the restenosis-free rate among the survivors was 88%.

Physiological and pathophysiological factors leading to restenosis were studied. Changes in the levels of two acute phase proteins, plasma fibrinogen, and serum C-reactive protein (s-CRP) were determined in 117 patients with severe carotid stenosis after eversion endarterectomy [63]. During the postoperative period sharp, highly significant drop occurred in the serum concentrations of both acute phase proteins. These findings indicate that the removal of atherosclerotic plaques from the carotid arteries markedly decreases the production of the two acute phase proteins. This phenomenon is due to the decrease of the inflammatory burden.

### 3.2. Role of Mannose-Binding Lectin and Complement on Restenosis

The role of mannose-binding lectin (MBL2) genotype in restenosis after eversion endarterectomy in patients with severe carotid atherosclerosis has been also studied [64]. MBL is thought to influence the pathophysiology of the cardiovascular system by decreasing the risk of advanced atherosclerosis and by contributing to enhanced ischemia reperfusion injury. Our data show that carotid duplex scan (CDS) values in patients homozygous for the normal (A) MBL2 genotype were significantly higher 14 months postsurgery as compared to the values measured 6 weeks after surgery. On the other hand, only a slight increase was registered in patients carrying variant MBL2 alleles. The frequency of MBL2 variant genotype was significantly higher in patient's not experiencing restenosis as compared with patients with restenosis. These results indicate that reoccurrence of stenosis after carotid endarterectomy is influenced by genetic factors and imply that MBL2 contributes to the pathophysiology of this condition.

In our previous study, we analyzed the relationship of C3 complement component with early restenosis, detected by CDS, after eversion endarterectomy by analysing three noncomplement acute-phase reactants (APRs) (C-reactive protein, haptoglobin, and alpha2-HS glycoprotein/fetuin-A) as control [65]. In MBL2 A/A allele carriers C3 levels increased during the follow-up period and correlated with the occurrence of restenosis at 14 months postsurgery. Even after adjusting for MBL2 genotype, age and gender, patients with high C3 levels had nearly five-fold higher odds for the development of significant restenosis (>50% reduction in diameter) By contrast, no such associations were detected between early restenosis and the noncomplement APRs. C3 is thus associated with the development of an early restenosis after eversion endarterectomy, which is related to an intact MBL lectin pathway. These results suggest that C3 levels probably have clinical importance and indicate that the regulation of C3 differs from noncomplement APRs.

Several growth factors are also suggested to contribute to restenosis. We investigated whether early postoperative changes in serum vascular endothelial growth factor (VEGF) and platelet-derived growth factor (PDGF) concentrations are in correlation with the MBL2 genotype in the context of the development of restenosis [66]. The data indicated that pronounced significant increases in both VEGF and PDGF-predicted restenosis exclusively in patients who were homozygous for the normal MBL2 genotype. In this group, the adjusted odds ratio of restenosis at 14 months was 27.7 (2.4 to 317.2) in patients with high early VEGF and PDGF increases, while in patients with low early growth factor increases the odds ratio was 9.2 (1.4 to 58.7). These findings indicate that in patients disposed to restenosis by MBL2 genotype pathologic processes leading to enhanced production of VEGF and PDGF during the very early postoperative period exert more harmful effect as compared to patients with the “protective” MBL2 genotype [66].

Considering the impact of MBL2 genotype on the occurrence of early restenosis after CEA, the role of C1-inhibitor (C1-INH), known as an inhibitor of the lectin pathway, has been also studied [67]. The aim of the study was to determine whether the C1-INH levels have predictive value for restenosis after CEA. Patients with >50% restenosis had significantly lower C1-INH levels at 6 weeks and at 4 days postsurgery. C1-INH levels at 6 weeks correlated inversely with the CDS values at 14 months (*r* = −0.3415), but only in MBL2 A/A homozygotes (*r* = −0.5044). Patients with low C1-INH levels had higher CDS values already at 7 months postsurgery. Patients with MBL2 A/A and low C1-INH levels at 6 weeks postsurgery had 13.9 times higher risk developing an early restenosis. These data indicate that MBL2 genotyping together with measuring the C1-INH level at 6 weeks postsurgery should be useful for identifying patients with high risk for early carotid restenosis.

## 4. Therapy

Pharmacological treatments currently in use include anticoagulants, antiplatelet agents, immunosuppressants, and antiproliferation agents. Unfortunately, none of these approaches has been conclusively shown to prevent coronary restenosis after balloon angioplasty or graft restenosis after peripheral arterial bypass. Consequently, the treatment of vascular stenosis remains today predominantly within the domain of the surgery. However, numerous successful animal experiments and promising proposals, as well as initial clinical results have been published lately. Here we review some remarkable contributions to this field.

### 4.1. Preclinical Investigations

As unfolded earlier, hypoxia is one of the risk factors which initiate the pathological mechanism leading to restenosis. The obvious target cells of restenosis therapy are the permanent proliferating VSMCs and the endothelial cells with injured function. The possibility of influencing the process of the phenotype switch of VSMCs to proliferative form during pathological, ischemic conditions would open up new vistas in the prevention and/or therapy of restenosis. Techniques addressing directly the proliferating VSMCs or injured endothelial cells have demonstrated significant degrees of clinical success.

A recent review has summarizied the present status of cellular and animal studies aimed at preventing restenosis by using protein kinase C (PKC) inhibitors. These agents are expected to exert therapeutically beneficial effects on restenosis causing factors of various kinds, including VSMCs [68].

Angiogenesis, that is, the growth of new blood vessels from existing host vessels, is recognized more and more as an important factor in the growth and progression of restenosis. Neovascularization as a pathological process was already known in the 19th century. The phenomenon occurs when the intima thickens beyond the diffusion limits of oxygen and nutrients, as a response to tissue hypoxia and consists of growth and extension of adventitial blood vessels into the intima [69]. Under hypoxic conditions the hypoxia-inducible factor alpha is upregulated and promotes hypoxia-dependent neovascularization. Blood vessels are destabilized, and this leads ultimately to vessel regression. In the presence of vascular endothelial growth factor (VEGF), Ang2 facilitates vascular sprouting with enhanced migration and proliferation of ECs. A high Ang2/Ang1 ratio has been found in vulnerable neovascularized plaques [70]. Other molecules and growth factors (VEGF and FGF) have also been shown to induce neovascularization and thereby increase nutrient perfusion. Treatment with these agents was named therapeutic angiogenesis by Freedman and Isner [71]. VEGF has therapeutic potential in cardiovascular gene therapy, the enhancement of arterioprotective endothelial functions, prevention postangioplasty restenosis. VEGF promotes the cell growth and survival, regeneration of endothelial cells. Whereas the production of NO in endothelial cells is induced by VEGF, the proliferation of vascular smooth muscle cell is inhibited. Inhibition of neointimal hyperplasia may also be achieved by gene transfer of endothelial NO synthase (eNOS), PGI synthase, or cell-cycle regulators cyclin-dependent kinase inhibitors, p53, and transcription factors such as nuclear factor kappaB [72]. The opposite effects are resulted by VEGF-inhibited angiogenesis in oncology.

It has been suggested that bone marrow-derived endothelial progenitor cells (EPCs) or circulating endothelial progenitor cells play a role in neovascularization [73, 74]. Nevertheless, it is not yet clear whether these progenitor cells should be considered as therapeutic targets in vascular diseases [75].

Iroxanadine (5,6-dihydro-5-(1-piperidinyl)methyl-3-(3-pyridil)-4H-1,2,4-oxadiazine) is a drug candidate with a marked cytoprotection by heat shock protein (HSP) coinduction. It has been originally synthesized by Biorex-Hungary (BRX-235) [76], and the worldwide rights have been recently sold by CytRx/California/USA to Orphazyme ApS/Novo A/S, Copenhagen, Denmark. In a recent work we have shown that BRX-235 stimulates the migration of ECs via p38 stress-activated-protein-kinase (SAPK) and enhanced expression of HSPs and heat-shock transcription factors [77]. After treatment by BRX-235, the vigorous proliferation of MIH cells decreased while the proliferation of deregulated EC cells increased. Simultaneously, the expression of both HSP72 and Cdkn1a was upregulated. This finding harmonizes with a clinical report demostrating compromised EC proliferation, and HSP72 expression was upregulated in VSMCs [78]. Neointima formation due to enhanced VMSC proliferation is suggested to be associated with reduced cyclic guanosine monophosphate signaling. We have undertaken to study the effect of the inhibition of cyclic guanosine monophosphate degradation on neointima formation in a rat model of endarterectomy, by using the selective phosphodiesterase-5 inhibitor vardenafil as a pharmacological inhibitor. The results were evaluated by conventional microscopy with hematoxylin and eosin staining and by immunohistochemical analysis to confirm neointima formation and the local cyclic guanosine monophosphate content [79]. Immunohistochemical analysis demonstrated intensive staining for transforming growth factor beta1 (TGF beta1) and alpha-smooth muscle actin in the control neointima. Vardenafil significantly reduced the stenosis grade (24.64% versus 54.12% in the control group) and expression of TGF beta1, as well as alpha-smooth muscle actin in the neointima. On the basis of these results it was suggested to consider the treatment with vardenafil as a new possibility to prevent neointimal hyperplasia after endarterectomy.

### 4.2. Clinical Observations

Stent insertion, one of the most common surgical techniques applied in vascular diseases, is too often followed by early, as well as late restenosis. To attenuate this complication, instead of conventional stents, drug-eluting stents (DESs) are more and more frequently applied [80].

Stents generally attenuate endothelial recovery, altering thereby the natural biology of the vessel wall and increasing the associated risk of stent thrombosis [81]. DESs, similarly to brachytherapy, target proliferating VSMCs at the site of injury and have been successful in reducing the formation of neointimal lesion [80–82]. DESs releasing various antiproliferative drugs represent also a useful strategy for the prevention of restenosis. VEGF-2 gene-eluting stents accelerate reendothelization, thereby offering an alternative strategy for the prevention of restenosis. This type of treatment, when applied in hypercholesterolemic rabbits, resulted in transgene expression in the vessel wall and a 2.4-fold increase in NO production by the ECs, along with improved functional recovery of stented segments [82].

The DES technique significantly decreases the incidence of restenosis. Follow-up studies, however, show the possible onset of late stent thrombosis [81]. Delayed arterial healing most probably associated with poor endothelialization of stent struts seems to be the underlying mechanism of this complication [83]. As recent results indicate, balloon angioplasty and stent implantation might still go hand in hand with severe postoperative complications. Several drugs have been suggests to use in DES, for example, antitumor agents (paclitaxel, doxorubicine, rapamycin, erythromycin, etc.), cholesterin lowering drugs (statins), immunosuppressants (cyclosporin), anti-inflammatory agents (Rapamycin), antibiotics (amphotericin B), and natural products (shikonin). Rapamycin is considered to be extremely useful in preventing restenosis [84].

### 4.3. Gene Therapy

Finally, we briefly summarize the achievements of gene therapy, as an emerging approach to counteract the pathological processes leading to restenosis. None of the gene therapy agents have been approved for clinical use up to now, but several promising animal experiments and preclinical studies have been recently published. The great potential advantage of the gene therapy resides in its potential to ensure specific, controlled expression of selected proteins within the targeted cell types. The panoply of gene therapy possibilities, among others the potential of promoting a functional epithelium, inhibiting SMC proliferation and therapeutic angiogenesis has been summarized in a review [85]. Several approaches of gene transfer have been designed for the treatment of postangioplasty restenosis. Adenoviruses (Ads) and adeno-associated viruses (AAVs) are currently the most efficient vectors for delivering therapeutic genes into the vascular system [86, 87]. Gene therapy is often associated with the drug-eluting stent (DES) technique; that is, the therapeutic gene is delivered by the stent [85, 87]. Along this line, local delivery of NOS gene seems to be the most promising approach. On the basis of successful animal application of iNOS gene transfer, a single-blind, multicenter clinical study has been recently performed by iNOS lipoplex gene therapy [88]. The results suggest that the technique applied provide a safe therapeutic principle for the prevention of coronary restenosis. Consequently, the authors suggest further clinical research to develop this promising possibility.

## 5. Conclusion

As a conclusion, we predict that significant clinical benefits are to be expected when using complex therapeutically approaches, which stimulate physical and functional recovery of the endothelial monolayer and in the same time inhibit the proliferation of vascular smooth muscle cells.
